# Somatic mutations reveal complex metastatic seeding from multifocal primary prostate cancer

**DOI:** 10.1002/ijc.34226

**Published:** 2022-08-09

**Authors:** Kristina T. Carm, Bjarne Johannessen, Mari Bogaard, Anne Cathrine Bakken, Aase V. Maltau, Andreas M. Hoff, Ulrika Axcrona, Karol Axcrona, Ragnhild A. Lothe, Rolf I. Skotheim

**Affiliations:** ^1^ Department of Molecular Oncology Institute for Cancer Research, Oslo University Hospital‐Radiumhospitalet Oslo Norway; ^2^ Institute for Clinical Medicine Faculty of Medicine, University of Oslo Oslo Norway; ^3^ Department of Pathology Oslo University Hospital‐Radiumhospitalet Oslo Norway; ^4^ Department of Urology Akershus University Hospital Lørenskog Norway; ^5^ Department of Informatics, Faculty of Mathematics and Natural Sciences University of Oslo Oslo Norway

**Keywords:** heterogeneity, multifocality, prostate cancer, targeted sequencing

## Abstract

Primary prostate cancer shows a striking intraorgan molecular heterogeneity, with multiple spatially separated malignant foci in the majority of patients. Metastatic prostate cancer, however, typically reveals more homogenous molecular profiles, suggesting a monoclonal origin of the metastatic lesions. Longitudinal mutational spectra, comparing multiple primary lesions with metastases from the same patients remain poorly defined. We have here analyzed somatic mutations in multisampled, spatio‐temporal biobanked lesions (38 samples from primary foci and 1 sample from each of 8 metastases from seven prostate cancer patients) applying a custom‐designed panel targeting 68 prostate cancer relevant genes. The metastatic samples were taken at time of primary surgery and up to 7 years later, and sampling included circulating tumor DNA in plasma or solid metastatic tissue samples. A total of 282 somatic mutations were detected, with a range of 0 to 25 mutations per sample. Although seven samples had solely private mutations, the remaining 39 samples had both private and shared mutations. Seventy‐four percent of mutations in metastases were not found in any primary samples, and vice versa, 96% of mutations in primary cancers were not found in any metastatic samples. However, for three patients, shared mutations were found suggesting the focus of origin, including mutations in *AKT1*, *FOXA1*, *HOXB13*, *RB1* and *TP53*. In conclusion, the spatio‐temporal heterogeneous nature of multifocal disease is emphasized in our study, and underlines the importance of testing a recent sample in genomics‐based precision medicine for metastatic prostate cancer.

## Graphical abstract

What's new?

Intrapatient tumor heterogeneity and clonal evolution of primary tumor foci into metastatic disease remain significant challenges for prostate cancer treatment. Here, investigating metastatic lesion origins, the authors compared somatic mutations in spatially distinct primary foci to mutations in recurring disease in seven prostate cancer patients. No overlap in mutations was identified between the majority of primary and metastatic lesions. Shared mutations, however, were found in three patients, revealing a possible metastasizing primary focus. Recurrent disease was marked by extensive temporally accumulated mutation burden. The findings highlight the utility of mutation analyses in better understanding molecular heterogeneity in prostate cancer.

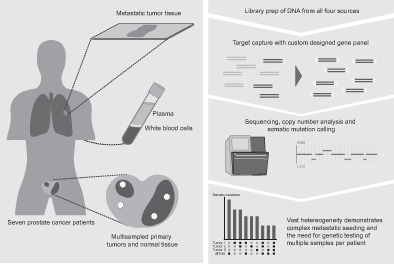

**Abbreviations**
cfDNA: cell‐free DNA
ctDNA: circulating tumor DNA
DNA: deoxyribonucleic acid
FFPE: formalin‐fixed paraffin‐embedded
PSA: prostate‐specific antigen
RNA: ribonucleic acid
SNP: single‐nucleotide polymorphism
ULP‐WGS: ultralow pass whole‐genome sequencing

## 1 INTRODUCTION

Prostate cancer is the second most frequent cancer type among men in the western world,^1^ and represents a major burden to healthcare and society. Upon diagnosis, patients are typically divided into three risk groups.^2^ However, the current risk group classification is coarse, and often not sufficient in a diagnostic setting. Routine PSA measurements have reduced overall mortality, but in parallel also an increased risk of overtreatment.^3^ Thus, molecular biomarkers that are able to distinguish the aggressive tumors from the indolent ones, and guide optimized tailored treatments, remains an unmet need.^4^

Several prognostic biomarkers have been explored,^5^ such as the presence of fusions involving *ERG*, mutations in DNA repair genes like *BRCA1*, *BRCA2* and *ATM*, and overexpression of the long noncoding RNA *SCHLAP1*.^6^

However, the development and implementation of specific biomarkers for prostate cancer is largely impeded by the multifocal nature of this disease. Several studies have elucidated the multifocal nature of prostate cancer, and as many as 60% to 90% of patients have multiple distinct primary tumor foci at the time of diagnosis.^7^ Substantial molecular heterogeneity, including nonoverlapping sets of somatic mutations, has been reported across these different tumor foci.^8^ Multiple tumor foci that represent contrasting molecular and prognostic signatures and thus levels of aggressiveness have been reported from within the same prostate.^9^, ^10^ Still, much of the tissue‐based research on prostate cancer investigates only the so‐called index tumor. Notably, the heterogeneity is most pronounced for primary tumors, whereas various metastatic lesions from the same patient have been shown to be more homogeneous,^11^, ^12^ indicating a common primary origin. Liquid biopsies have been used to track various metastatic routes in prostate cancer patients.^13^

In the present study, we aim to distinguish the aggressive primary focus from the indolent ones, and further to explore the mutational relationship with the metastatic disease.

## 2 MATERIALS AND METHODS

### 2.1 Patient material

Seven prostate cancer patients who underwent radical prostatectomy between 2010 and 2012 at Oslo University Hospital‐*Radiumhospitalet*, and whom have developed metastases up to 7 years after surgery were included in the study. From three of these seven patients (Patients 1‐3), metastatic samples (one lung‐, and two pelvic lymph node metastases) were obtained from formalin‐fixed paraffin‐embedded (FFPE) tissue blocks from surgically removed metastases (one from lung and two from lymph nodes).

For Patients 3 to 7, blood samples with an elevated PSA level were included to capture potential mutations in an early phase of the metastatic process. These patients' diseases were represented by circulating cell‐free DNA (cfDNA) (harvested 41‐91 months after prostatectomy). The blood samples were selected from a cohort of 40 patients with blood samples with an elevated PSA level (>0.2 ng/mL) due to the presence of clearly multifocal cancer and available tissue samples from multiple foci (Patients 3‐7, Supplementary Table S1). Blood samples from these 40 patients were collected at various time points after prostatectomy with a median of 53 months after surgery. Plasma was separated from whole blood immediately after blood collection, and stored at −80°C until isolation of cfDNA was performed.

Fresh frozen or FFPE primary tissue was available from all patients from either two or three tumor foci. From each patient, one corresponding normal (peripheral blood) or benign (FFPE tissue) DNA sample was included for use as reference in somatic variant calling. Clinicopathological data on all seven patients and number and types of samples are summarized in Supplementary Tables S2 and S3.

DNA from all included samples was isolated using the AllPrep DNA/RNA/miRNA Universal Kit, AllPrep DNA/RNA FFPE Kit, QIAamp MinElute ccfDNA Mini kit or QIAamp DNA Blood Maxi kit (Qiagen, Venlo, the Netherlands) according to the manufacturer's protocol.

### 2.2 DNA sequencing for determination of tumor fraction of cfDNA and targeted sequencing

To obtain DNA sequence information from all included samples from prostate cancer patients, a targeted prostate cancer‐relevant sequencing panel was designed based on available gene lists from other publications. A detailed description of the design process, inclusion criteria and the final gene list is included in Supplementary methods, Supplementary Figure S1 and Supplementary Table S4.

DNA sequencing libraries were prepared from 10 to 20 ng of cfDNA or 250 ng of DNA from white blood cells or tissue samples using the KAPA HyperPlus kit (Roche, Basel, Switzerland) with xGen Dual Index UMI Adapters from Integrated DNA Technologies (Coralville, IA) according to the manufacturers protocol.

Ultralow pass whole‐genome paired end (2 × 75 bp) sequencing (ULP‐WGS) was performed NextSeq 550 sequencing system (Illumina, San Diego, CA), with a mean sequencing depth of 0.3X on all cfDNA samples to determine the fraction of circulating tumor DNA in cfDNA (Supplementary Figure S2). For a detailed description of the analysis after sequencing, see Supplementary methods.

For target capture, the Twist Target Enrichment Protocol (Twist Bioscience, San Francisco, CA) was applied according to the manufacturer's protocol. Target captured libraries were applied to the MiniSeq sequencing system (Illumina) using the MiniSeq High Output Reagent Kit (150 cycles, Illumina). For Patients 5 to 7, raw alignment reads were obtained using a whole exome sequencing protocol, and the preprocessing of tumor bam files was performed as previously described.^8^ See Supplementary methods for a detailed description of the variant calling. The sequencing coverage and quality statistics for ULP‐WGS and targeted sequencing for each sample are summarized in Supplementary Tables S1 and S3.

### 2.3 Patient identity matching after sequencing of included DNA samples

To verify matching patient identities between all primary and metastatic samples, we applied the SAMtools (version 1.8) mpileup command on all SNPs from dbSNP (version 150) within the captured regions.^14^ Using only those SNPs for which all samples where covered by a minimum of 10 reads, and with at least one sample having at least 10% variant allele frequency, 390 SNPs were identified. Principal components analysis provided satisfactory demonstration that all samples from each of the seven patients clustered together and apart from all other patients (Supplementary Figure S3).

## 3 RESULTS

Targeted DNA sequencing was performed on metastatic prostate cancer and patient‐matched tissue samples from radical prostatectomies of seven patients, using a custom‐made prostate cancer‐focused gene‐panel.

**FIGURE 1:**
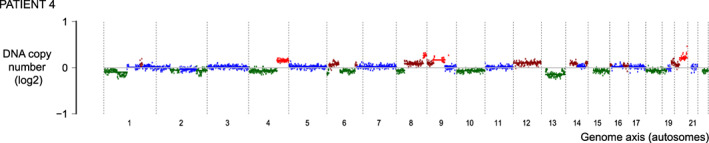
Genome‐scale DNA copy numbers from cell‐free DNA (cfDNA). Analysis of data from ultralow pass whole‐genome sequencing of cfDNA from Patient 4 yields an estimated tumor‐derived fraction (ctDNA) of 11%. The plot is restricted to autosomes. Plots from four additional cfDNA samples are found in Supplementary Figure S2

Four of the patients included in the targeted analysis were selected from a series of 40 patients from whom cfDNA was explored by genome‐scale copy numbers to estimate the fraction of ctDNA (tumor content and PSA‐level in all 40 blood samples are listed in Supplementary Table S1). Median PSA level among the 40 samples was 0.5 ng/mL (range 0.2‐39 ng/mL). Notably, only six of the patients had a tumor content above 3%, and only one of the patients with PSA >1 ng/mL had a tumor content above 2%. Patients 3 to 7 (Figure 1, Supplementary Figure S2, and Supplementary Table S1) were selected for targeted analysis due to the availability of samples from two or more foci in addition to cfDNA.

From the altogether seven patients, and 46 samples, a total of 282 somatic point mutations were detected in 49 of 68 targeted genes (Supplementary Table S5). The range of somatic mutations per sample was between 0 and 25. Well‐known cancer critical genes like *BRCA1*, *IDH1*, *ERG*, *KMT2C*, *MSH6* and *PALB2* were among the mutated genes in the metastatic samples. Interestingly, none of these genes were found to be mutated in any of the corresponding primary malignant samples. In total, 151 unique mutations were detected; 110 of these were exclusively called from primary tumors or benign areas, 31 exclusively from metastatic disease (tumor or cfDNA), whereas 10 were shared between samples from primary and metastatic sites.

**FIGURE 2:**
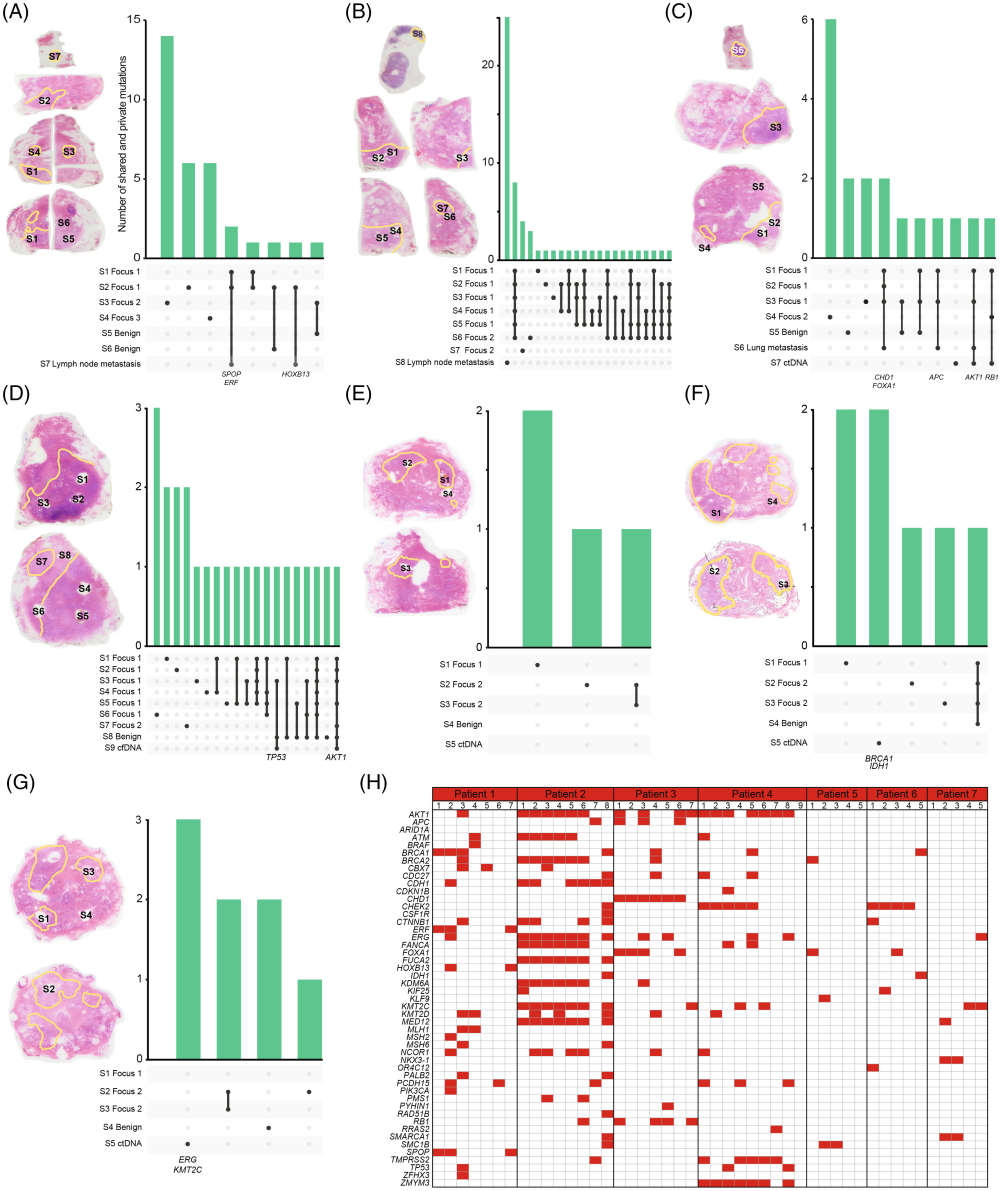
Sample origin and number of shared and private somatic point mutations in four prostate cancer patients. (A) Patient 1, (B) Patient 2, (C) Patient 3, (D) Patient 4, (E) Patient 5, (F) Patient 6 and (G) Patient 7. Samples were taken from several different parts of the radical prostatectomies as illustrated with hematoxylin‐eosin stained tissue sections to the left of each figure. In A‐C, the metastasis is depicted first, with the sections from the radical prostatectomy below. In D‐G, only sections from the primary prostate cancer lesions are shown. (H) Mutated genes across all included samples. Only genes in which somatic mutations were detected are included, and intragene positions may differ. Detailed information on specific mutations can be found in Supplementary Table S5

An overview of all shared and private mutations for each of the seven patients are given in Figure 2. Altogether 36 of the 46 samples shared a somatic mutation with at least one other sample from the same patient. Thirty of the 46 samples had private somatic mutations and only 21 out of 282 mutations were shared between different cancer foci in the same patient (primarily in Patient 2).

Specifically, in Patient 1, somatic point mutations in the genes *SPOP*, *ERF* and *HOXB13* were found to be shared between the lymph node metastasis and two (S1 and S2) out of three primary malignant samples from Focus 1.

In Patient 2, there were no shared mutations between the primary and metastatic samples (Figure 2).

In Patient 3, samples from Focus 1 and the lung metastasis shared the same mutations in *CHD1*, *FOXA1* and *APC*, and a mutation in *RB1* was shared between ctDNA and samples from both primary foci (Figure 2). One mutation, in *AKT1*, was found to be shared between the two metastatic samples as well as samples from Focus 1. Whereas the lung metastasis matched only Focus 1, the cfDNA had one somatic mutation in common with Focus 1, and another in common with Focus 2. Both these mutations were reliably called, and points to a double metastatic seeding from this patient.

Reevaluation of tissue slides from Patients 1, 2 and 3 revealed that the samples had varying tissue morphology (Supplementary Figure S4 and Supplementary results). The metastatic sample in Patient 1 showed similar morphology as Focus 1, which was also true for the metastatic sample in Patient 3. In Patient 2, the metastasis showed a morphology not identified in the surrounding area of the primary samples.

In Patient 4, one shared mutation in *AKT1* was found in both the cfDNA and in samples from Focus 1 (S1, S2, S3 and S5), Focus 2 (S7) and the benign tissue sample (S8). Interestingly, one mutation in *TP53* was detected in cfDNA, Focus 1 (S3) and the benign sample (S8).

In the remaining patients (Patients 5‐7), no shared mutations were found between the metastatic sample (ctDNA) and the primaries. In Patient 5, ctDNA harbored no detected mutations in the included genes. Conversely, ctDNA from both Patients 6 and 7 had mutations in two known cancer critical genes each (*BRCA1* and *IDH1*, and *ERG* and *KMT2C*, respectively).

## 4 DISCUSSION

In the present study, we found extensive spatio‐temporal diversity of mutational profiles across samples from all included patients. Among 68 prostate cancer relevant genes, shared somatic mutations between primary foci and a metastatic lesion were found for three of the seven patients, indicating the origin of the metastatic clonal development. These results confirm the aggressiveness‐superiority of one focus over the others.^11^, ^15^ Studies have shown that over 20% of local metastatic disease lack shared alterations with the index lesion in the primary prostatectomy,^16^ which is somewhat lower than our results from multiple primary samples. Different metastatic lesions from the same patient have been shown to share molecular alterations,^11^, ^17^, ^18^, ^19^ as we demonstrate in one of our patients, where both cfDNA and a lung metastasis share a mutation in *AKT1*, but each of the samples also have two or three private mutations. It is assumed that cfDNA is heterogeneity ignorant, implying that the mutation spectrum found here represents the overall metastatic disease. This is indicated for Patient 4, since the cfDNA contain mutations found in both primary foci and in the lung metastasis (AKT1 and RB1). Intrafocal heterogeneity was observed in most patients, with samples from the same malignant focus harboring private mutations. Mutations in genes involved in homologous recombination repair, was found to be mutated in the majority of our patients. For example, mutation in *BRCA1* or *BRCA2* was found in one or more samples from six out of the seven patients. Therapeutic agents like PARP‐inhibitors have been shown to have effect in some prostate cancer patients with mutated homologous recombination repair pathways.^20^, ^21^ However, the heterogeneity we see across different malignant samples from the same patient challenges the detection of such mutations, and thus the introduction of genomics‐based personalization of medical oncologic treatment for prostate cancer.

For three of the four patients from which no shared mutations were found between the metastatic sample and any of the samples from their primary foci, the primary samples were analyzed using a previously generated exome sequencing dataset.^8^ Here, the sequencing depth was lower as compared to the targeted sequencing (Supplementary Table S3), and mutations with low variant allele frequencies might have been missed. Additionally, we may speculate that this reflects a temporal, evolutionary heterogeneity in these patients.^22^, ^23^ Even though some cells from one of the primary tumors have escaped the prostate capsule and grown outside of the prostate gland, these metastatic lesions are likely to have acquired private mutations. Further, since primary prostate cancers have relatively few somatic mutations compared to most other cancer types,^24^ evidence for the direct ancestral relation between metastatic and primary tumors may not exist within our prostate cancer specific gene panel of 68 genes, but may exist elsewhere in the genome. However, the genes in the current panel were selected to have a significant amount of somatic mutations in prostate cancer, as seen from nine studies performing genome‐scale mutation detection (Supplementary methods and Supplementary Table S4).

From one patient, mutations in *AKT1* and *TP53* were found both in cfDNA and benign and malignant samples. This may reflect a somatic mutation early in prostatic development. Also others have reported somatic mutations in cancer‐critical genes in benign prostate tissue.^25^ However, we cannot exclude that this result is due to invasion of malignant cells into benign‐appearing areas of the prostate or circulating tumor cells in the blood vessels surrounding normal prostatic tissue.

Interestingly, Patients 3 and 4, both with distant metastasis (Supplementary Table S2) shared the same mutation in *AKT1*, a previously reported hot spot mutation in prostate cancer.^26^ This proves how the use of targeted sequencing panels like the one we have designed can be utilized to trace the origin of aggressive disease, in accordance with a recent publication.^27^

Liquid biopsies, like the cfDNA investigated here, have been proposed as snapshots of present disease at the time of the blood withdrawal. From Patients 3 and 4, the included blood plasma sample was drawn 6 and 7 years after radical prostatectomy (PSA = 7.2 and 6.8 ng/mL, respectively). High levels of PSA after multiple time‐points with undetectable PSA long after the radical prostatectomy, points toward a more aggressive disease which has been rather dormant over time. However, somewhat surprising, but in line with what others have reported, relatively high levels of PSA is not correlated with high content of ctDNA (Supplementary Table S1).^28^

An important limitation of the current study is the relatively low number of patients included. However, to our knowledge, the present study is the only study combining both cfDNA, metastatic and primary tissue samples from multiple distinct tumor foci. This multisampling approach has provided important results on the spatial and also temporal heterogeneity in multifocal prostate cancer, including its complex metastatic seeding.

In conclusion, highly divergent sets of somatic mutations were found across a set of samples representing primary and metastatic prostate cancer. The vast both spatial and temporal heterogeneity clearly demonstrate that any somatic mutation based personalized medicine approach on metastatic prostate cancer benefits from analysis of the most recent sample.

## AUTHOR CONTRIBUTIONS

**Kristina T. Carm**: Planning, formal analysis, data curation, investigation, writing—original draft, visualization. **Bjarne Johannessen**: Formal analysis, resources, data curation, writing—original draft, visualization. **Mari Bogaard**: Investigation, writing—review & editing. **Anne Cathrine Bakken**: Investigation, writing—review & editing. **Aase V. Maltau**: Resources, writing—review & editing. **Andreas M. Hoff**: Conceptualization, resources, writing—review & editing. **Ulrika Axcrona**: Conceptualization, supervision, project administration, funding acquisition, resources, writing—review & editing. **Karol Axcrona**: Conceptualization, supervision, project administration, funding acquisition, resources, writing—review & editing. **Ragnhild A. Lothe**: Conceptualization, supervision, project administration, funding acquisition, writing—review & editing. **Rolf I. Skotheim**: Conceptualization, supervision, project administration, funding acquisition, writing—review & editing.

The work reported in the paper has been performed by the authors, unless clearly specified in the text.

## FUNDING INFORMATION

The study was funded by the South‐Eastern Norway Regional Health Authority (grant numbers 2017045, 2019016 and 2020063), the Research Council of Norway (grant numbers 262529/F20 and 250993) and the Norwegian Cancer Society (208197), and from the Anders Jahre Foundation for the Promotion of Science. The study was granted secure storage of computer files and high‐performance computation resources from NorStore and University of Oslo's Services for Sensitive Data (NS9013S).

## CONFLICT OF INTEREST

The authors declare no competing interests.

## ETHICS STATEMENT

Written informed consent was obtained from all included patients and the study was approved by the Regional Ethics Committee South‐Eastern Norway (number 2013/595/REK southeast A).

## Supporting information


**Appendix S1**. Supporting Information

**Supplementary Table S1**. Patients and blood samples included in ULP‐WGS analysis of cfDNA. Forty blood samples from 40 patients with biochemical relapse were included in ultralow pass whole genome sequencing (ULP‐WGS). The tumor fraction was estimated from copy number analysis after ULP‐WGS. PSA, prostate specific antigen
**Supplementary Table S2**. Clinical data for included patients, including information on recurrence and pathological stages, grade group and therapies. BCR, biochemical recurrence; cT, clinical tumor stage; ISUP, International Society of Urological Pathology; N stage, lymph node stage; PSA, prostate specific antigen; pT, pathological tumor stage
**Supplementary Table S3**. Samples included in the analyses
**Supplementary Table S4**. Genes included in gene panel for targeted sequencing, including capture size and selection criteria. Genes of interest was chosen after a literature review of relevant papers on both metastatic and primary prostate cancer (more information about the selection of included genes can be found in Supplementary methods). CN, copy number; CNA, copy number aberration
**Supplementary Table S5**. Detected somatic mutations

## Data Availability

The data that support the findings of our study are available from the corresponding author, Rolf I. Skotheim, upon reasonable request.
