# Supplementary material for: Somatic mutations reveal complex metastatic seeding from multifocal primary prostate cancer
Source: Int J Cancer. 2022 Aug 9;152(5):945–51. doi: 10.1002/ijc.34226 (PMC10087486; doi:10.1002/ijc.34226)
Supplement: Supplementary file 1 — Appendix S1. Supporting Information [file IJC-152-945-s001.pdf]

Somatic mutations reveal complex metastatic seeding from multifocal primary prostate cancer

Kristina Totland Carm, Bjarne Johannessen, Mari Bogaard, Anne Cathrine Bakken, Aase V. Maltau, Andreas M. Hoff, Ulrika Axcrona, Karol Axcrona, Ragnhild A. Lothe, and Rolf I. Skotheim

Table of Contents

Supplementary materials & methods 2

Supplementary results 5

Supplementary tables – available in separate files 6

Supplementary figures 7

Supplementary references 11

Supplementary materials & methods

Design of a prostate cancer-focused targeted sequencing panel

In total, the design consists of 2470 probes covering 243,706 base pairs, targeting 68 genes. The background information includes gene sets from significantly mutated genes in publications on both primary, and some including metastatic, prostate cancer. All these publications included analysis of at least 50 primary cancers, all generated using genome or exome-scale DNA sequencing [1-8]. In addition, a list of significantly mutated genes from an in-house analysis of multifocal primary prostate cancer was included[9]. Identities of the included genes, lengths of their exonic sequences, and which selection criteria they meet are listed in Supplementary Table S4. In short, twenty-six of the included genes were listed as significantly mutated in three or more of the chosen publications; ten were listed as significantly mutated in only two of the publications, but had a frequency of more than one mutation per million basepairs (Mbp); sixteen were listed as significantly mutated in only one of the chosen publications, but with a mutation frequency above three per Mbp; nine are found to be frequently mutated in the germline of hereditary prostate cancer [10-12]; and eight are DNA repair genes [13-16] which were added to detect mutations in this particularly clinically relevant system. Probes covering nine relevant genes were included to detect common DNA copy number aberrations commonly amplified or deleted in prostate cancer [9]. As the fusion gene *TMPRSS2-ERG* is present in around 50 % of all prostate cancers and commonly caused by a deletion of a 2.8 Mb region on chromosome 21 [17, 18], probes were designed to enable detection of this deletion/fusion. To detect DNA copy number changes in the enhancer region of *AR*; a recurrent event in metastatic prostate cancer, four probes were designed to capture this particular region [19]. To enable detection of more wide-spread DNA copy number changes, one probe was designed to target sequences in close proximity to each chromosome arm. An overview of all included regions can be found in Supplementary Table S4.

Ultra-low pass whole-genome sequencing of DNA for determination of tumor fraction of cfDNA

Raw sequencing reads were aligned to the hg19 reference genome using BWA (version 0.7.17). Alignment maps were sorted and converted to binary alignment files using Samtools.

The readCounter application from the Titan toolbox was applied to generate wig files based on the alignment bam files. From the wig files, the ichorCNA software was applied to determine the fraction of ctDNA in cfDNA 20 (DNA copy number plots and estimated tumor fractions can be found in Figure 1, Supplementary Figure S2 and Supplementary Table S1). Blood samples from patients with both available cfDNA and tissue samples from multiple foci were included in further analysis. The number of uniquely mapped reads for each sample was calculated using the command:

samtools view -c -L hg19.bed -f 1 -F 1284 -q 20 file.bam,

where the hg19 bed file was created from the same hg19 genome reference file as was used for raw read alignment.

Additional metrics presented in Suppl. Table S1 were calculated using the CollectWgsMetrics from Picard (version 2.6.0).

The ichorCNA software was used to determine the fraction of ctDNA in cfDNA [20] (DNA copy number plots and estimated tumor fractions can be found in Figure 1, Supplementary Figure S2 and Supplementary Table S1). Blood samples from patients with both available cfDNA and tissue samples from multiple foci were included in further analysis.

Targeted sequencing of DNA and mutation calling

After sequencing, fastq files were created from raw basecalls using tools from the Picard (version 2.19.0) toolbox. Barcodes were identified with the ExtractIlluminaBarcodes tool, and unaligned bam files with unique molecular indexing (UMI) information were created using the IlluminaBasecallsToSam algorithm. Fastq files were obtained by applying the SamToFastq tool, and

these raw reads were aligned to the hg38 genome reference using BWA (version 0.7.17). Sam files were coordinate-sorted and subsequently converted to binary format by the SortSam tool. Unaligned bam files with UMI information and aligned bam files without UMI information were merged using the MergeBAMAlignment tool. All bam files were subject to GATK (version 4.1.2.0) preprocessing.

Variant calling after sequence alignment and preprocessing, as described in the previous section, was performed using both the MuTect (SNVs), Strelka (indels), and MuTect2 (SNVs + indels) algorithms. Indels needed to be nominated by both indel callers to be included. Variants covered by one or more alternative reads in the normal sample and less than two alternative reads in the tumor were rejected. In addition, a cut-off of at least 1 % variant allele frequency (VAF) in the tumor sample was used. All mutations needed to be scored as 'PASS' in at least one sample to be included. Candidate variants in repetitive and non-complex genomic regions were discarded after visual inspection. Sequencing depth was calculated by taking the mean coverage across all exonic regions from 68 target genes using the DepthOfCoverage module from GATK (version 4.1.6.0). Total number of uniquely mapped non-duplicate reads was calculated using the following commands:

samtools view -c -L targeted_regions.bed -f 1 -F 1284 -q 20 file.bam. Other quantification measurements, including "Total number of covered targeted bases", "Median coverage (and range) per targeted base" and "Percentage of targeted bases with coverage >200" were calculated using the DepthOfCoverage module from GATK (version 4.1.6.0).

Patient identity matching of included DNA samples

To verify matching patient identities between all primary and metastatic samples, we ran the samtools (version 1.8) mpileup command on all SNPs from dbSNP (version 150) within the captured regions [21]. The analysis included the 390 SNPs for which all samples were covered by a minimum of 10 reads, and at least one sample had a 10 % variant allele frequency. Principal components analysis demonstrated that all samples from each of the four patients clustered together and apart from all samples of other patients (Supplementary Figure S3).

Supplementary results

Re-evaluation of tissue slides from patients with metastatic tissue samples

For Patient 1, the areas surrounding the samples from primary malignant tumor Focus 1 were composed of primarily of a Gleason grade 4 component with ill-defined, glomeruloid- and cribriform glands in addition to a small component of Gleason grade 3 (Supplementary Figure S4A). The sample from the metastasis showed a similar morphology as found in Focus 1, although due to thermal damage was to some extent difficult to evaluate. Focus 2 and 3 were composed of well-defined glands consistent with Gleason grade 3 (Supplementary Figure S4A).

For Patient 2, the area surrounding the samples from primary malignant tumor Focus 1 were composed primarily of a Gleason grade 4 component with ill-defined glands in addition to a small Gleason grade 3 and a Gleason grade 5 component (Supplementary Figure S4B). The sample from the metastasis was composed of cribriform glands consistent with Gleason grade 4, which was not identified in the surrounding area of the primary samples; cribriform glands were, however, identified in other areas of Focus 1. The primary samples from Focus 2 were composed solely of well-defined glands consistent with Gleason grade 3.

For Patient 3, the area surrounding the samples from primary malignant tumor Focus 1 were composed of a Gleason grade 4 component with primarily cribriform glands and a Gleason grade 3 component with well-defined glands (Supplementary Figure S4C). The sample from the metastasis was composed of cribriform glands, which was similar to the morphology found in the samples of Focus 1. Focus 2 displayed primarily Gleason grade 3 with a small component of Gleason grade 4. Cribriform glands were not identified in Focus 2.

Supplementary tables

The following supplementary tables are available as separate files.

Supplementary Table S1: Patients and blood samples included in ULP-WGS analysis of cfDNA.

Supplementary Table S2: Clinical data for included patients, including information on recurrence and pathological stages, grade group and therapies

Supplementary Table S3: Samples included in the analyses

Supplementary Table S4: Genes included in gene panel for targeted sequencing, including capture size and selection criteria

Supplementary Table S5: Detected somatic mutations.

Supplementary figures

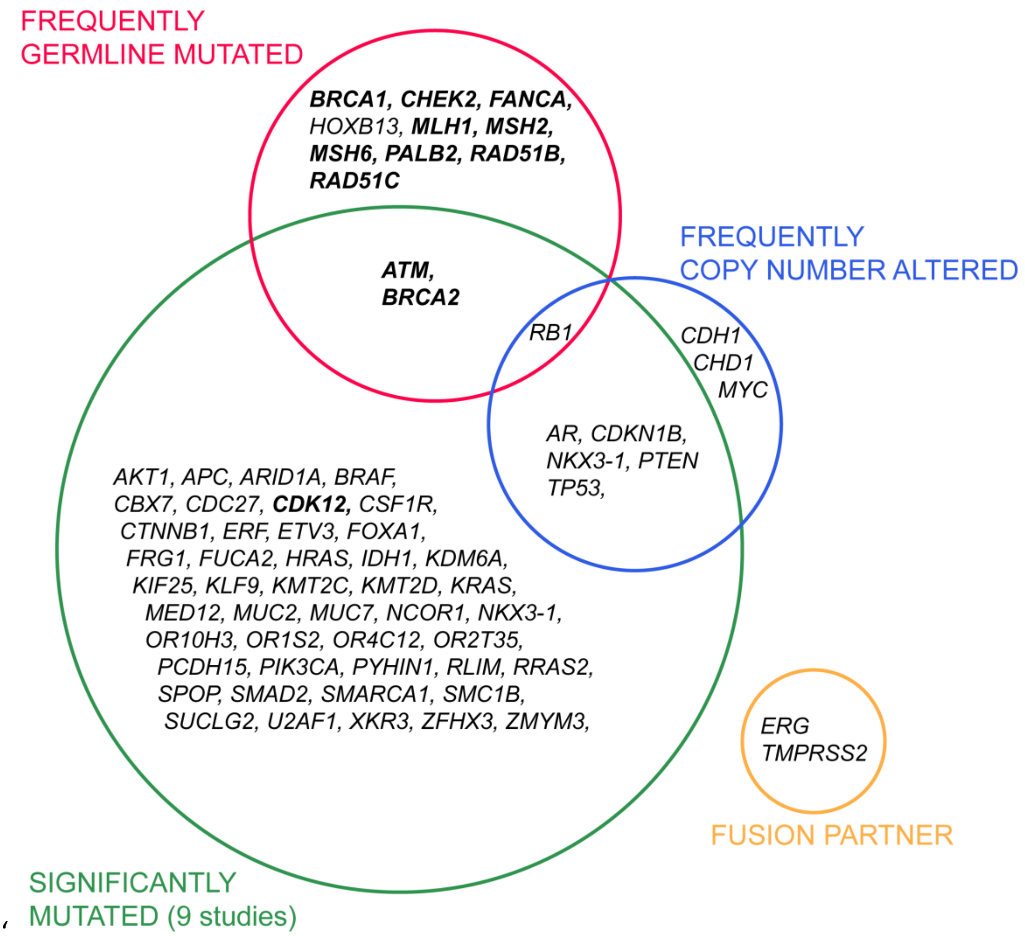

Supplementary Figure S1. Overview of the inclusion criteria for the 68 genes captured with the targeted sequencing panel.

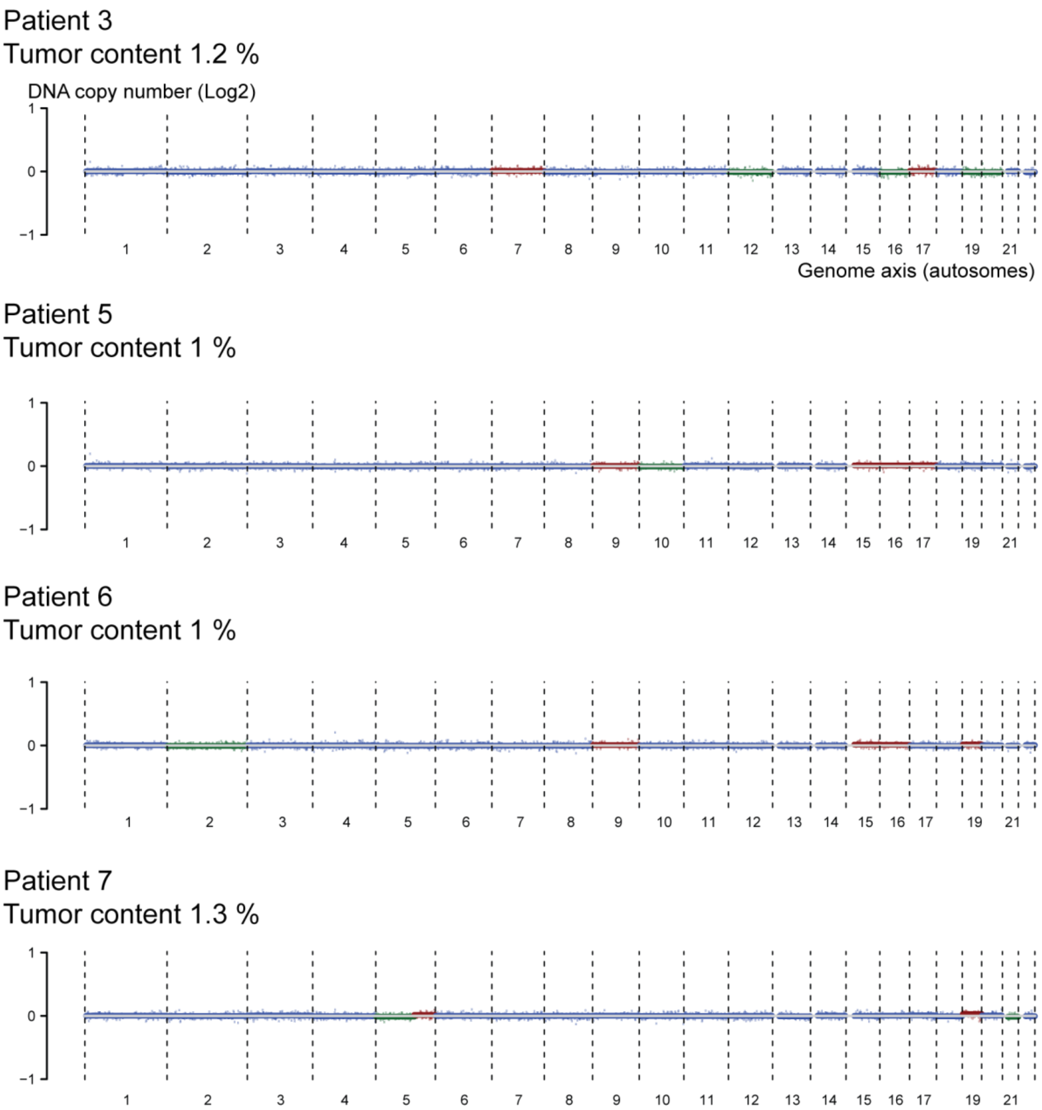

Supplementary Figure S2. DNA copy number profiles from circulating cell free DNA of patients 3, 5, 6 and 7 with metastatic prostate cancer. The raw data were generated with ultra-low pass whole-genome sequencing, and DNA copy number estimates and fraction of tumor-derived DNA were estimated using the ichorCNA software, as described in Supplementary methods.

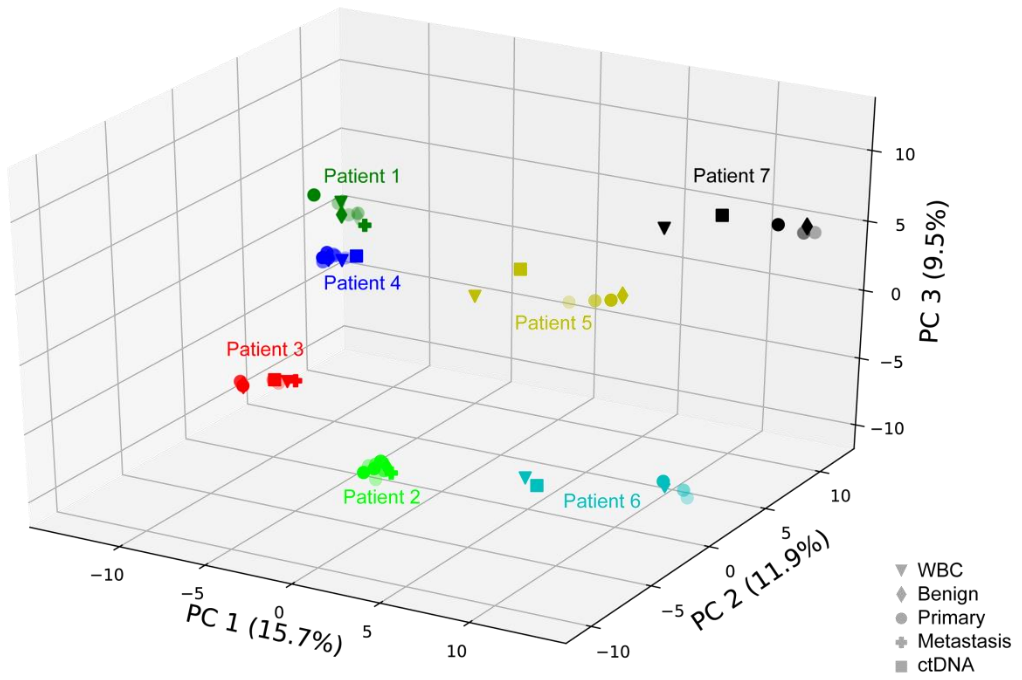

Supplementary Figure S3. Sample clustering per patient based on genotypes in polymorphic loci. Since several samples derived from the same patients had none or few shared somatic mutations, we used principal components analysis of genotype data from single nucleotide polymorphisms as a quality control to demonstrate that the samples derived from the same individual.

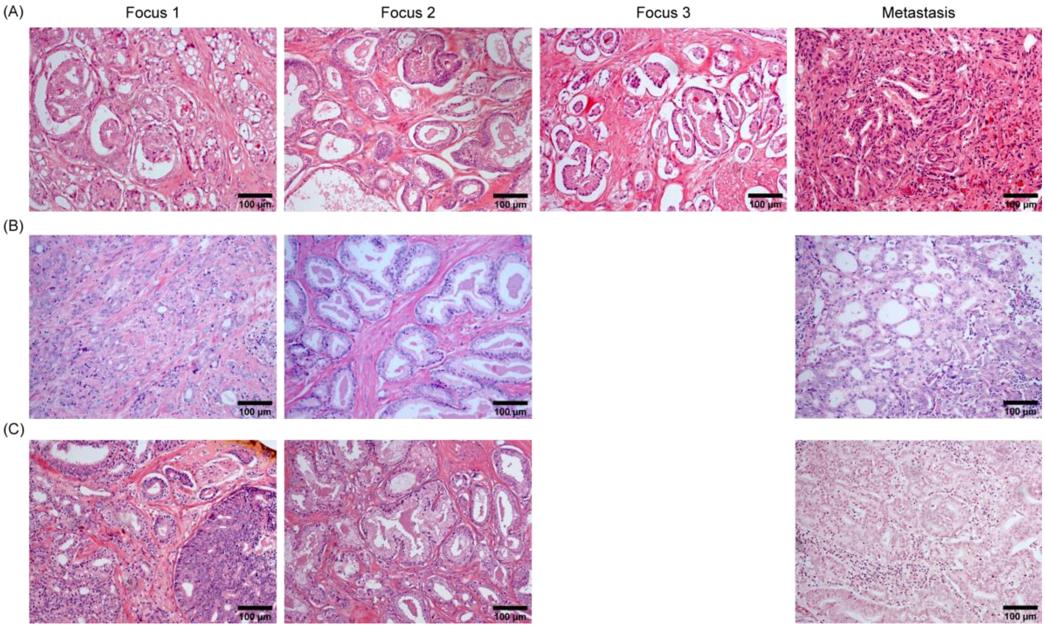

Supplementary Figure S4. Representative histology from hematoxylin and eosin stained tissue sections from (A) Patient 1, (B) Patient 2 and (C) Patient 3. (10X microscopy images). For all three patients, all present foci (two or three) and one metastatic sample are included.

Supplementary references

1. Armenia J, Wankowicz SAM, Liu D, Gao J, Kundra R, Reznik E, Chatila WK, Chakravarty D, Han GC, Coleman I, Montgomery B, Pritchard C, Morrissey C, Barbieri CE, Beltran H, Sboner A, Zafeiriou Z, Miranda S, Bielski CM, Penson AV, Tolonen C, Huang FW, Robinson D, Wu YM, Lonigro R, Garraway LA, Demichelis F, Kantoff PW, Taplin ME, Abida W, Taylor BS, Scher HI, Nelson PS, de Bono JS, Rubin MA, Sawyers CL, Chinnaiyan AM, Team PSCIPCD, Schultz N, Van Allen EM. The long tail of oncogenic drivers in prostate cancer. Nat Genet 2018;50:645-51.

2. Cancer Genome Atlas Research N. The Molecular Taxonomy of Primary Prostate Cancer. Cell 2015;163:1011-25.

3. Barbieri CE, Baca SC, Lawrence MS, Demichelis F, Blattner M, Theurillat J-P, White TA, Stojanov P, Van Allen E, Stransky N, Nickerson E, Chae S-S, Boysen G, Auclair D, Onofrio RC, Park K, Kitabayashi N, MacDonald TY, Sheikh K, Vuong T, Guiducci C, Cibulskis K, Sivachenko A, Carter SL, Saksena G, Voet D, Hussain WM, Ramos AH, Winckler W, Redman MC, Ardlie K, Tewari AK, Mosquera JM, Rupp N, Wild PJ, Moch H, Morrissey C, Nelson PS, Kantoff PW, Gabriel SB, Golub TR, Meyerson M, Lander ES, Getz G, Rubin MA, Garraway LA. Exome sequencing identifies recurrent SPOP, FOXA1 and MED12 mutations in prostate cancer. Nature Genetics 2012;44:685.

4. Fraser M, Sabelnykova VY, Yamaguchi TN, Heisler LE, Livingstone J, Huang V, Shiah Y-J, Yousif F, Lin X, Masella AP, Fox NS, Xie M, Prokopec SD, Berlin A, Lalonde E, Ahmed M, Trudel D, Luo X, Beck TA, Meng A, Zhang J, D'Costa A, Denroche RE, Kong H, Espiritu SMG, Chua MLK, Wong A, Chong T, Sam M, Johns J, Timms L, Buchner NB, Orain M, Picard V, Hovington H, Murison A, Kron K, Harding NJ, P'ng C, Houlahan KE, Chu KC, Lo B, Nguyen F, Li CH, Sun RX, de Borja R, Cooper CI, Hopkins JF, Govind SK, Fung C, Waggott D, Green J, Haider S, Chan-Seng-Yue MA, Jung E, Wang Z, Bergeron A, Dal Pra A, Lacombe L, Collins CC, Sahinalp C, Lupien M, Fleshner NE, He HH, Fradet Y, Tetu B, van der Kwast T, McPherson JD, Bristow RG, Boutros PC. Genomic hallmarks of localized, non-indolent prostate cancer. Nature 2017;541:359.

5. Lindberg J, Mills IG, Klevebring D, Liu W, Neiman M, Xu J, Wikström P, Wiklund P, Wiklund F, Egevad L, Grönberg H. The Mitochondrial and Autosomal Mutation Landscapes of Prostate Cancer. European Urology 2013;63:702-08.

6. Ren S, Wei G-H, Liu D, Wang L, Hou Y, Zhu S, Peng L, Zhang Q, Cheng Y, Su H, Zhou X, Zhang J, Li F, Zheng H, Zhao Z, Yin C, He Z, Gao X, Zhau HE, Chu C-Y, Wu JB, Collins C, Volik SV, Bell R, Huang J, Wu K, Xu D, Ye D, Yu Y, Zhu L, Qiao M, Lee H-M, Yang Y, Zhu Y, Shi X, Chen R, Wang Y, Xu W, Cheng Y, Xu C, Gao X, Zhou T, Yang B, Hou J, Liu L, Zhang Z, Zhu Y, Qin C, Shao P, Pang J, Chung LWK, Xu J, Wu C-L, Zhong W, Xu X, Li Y, Zhang X, Wang J, Yang H, Wang J, Huang H, Sun Y. Whole-genome and Transcriptome Sequencing of Prostate Cancer Identify New Genetic Alterations Driving Disease Progression. European Urology 2018;73:322-39.

7. Abida W, Armenia J, Gopalan A, Brennan R, Walsh M, Barron D, Danila D, Rathkopf D, Morris M, Slovin S, McLaughlin B, Curtis K, Hyman DM, Durack JC, Solomon SB, Arcila ME, Zehir A, Syed A, Gao J, Chakravarty D, Vargas HA, Robson ME, Joseph V, Offit K, Donoghue MTA, Abeshouse AA, Kundra R, Heins ZJ, Penson AV, Harris C, Taylor BS, Ladanyi M, Mandelker D, Zhang L, Reuter VE, Kantoff PW, Solit DB, Berger MF, Sawyers CL, Schultz N, Scher HI. Prospective Genomic Profiling of Prostate Cancer Across Disease States Reveals Germline and Somatic Alterations That May Affect Clinical Decision Making. JCO precision oncology 2017;2017:10.1200/PO.17.00029.

8. Wedge DC, Gundem G, Mitchell T, Woodcock DJ, Martincorena I, Ghori M, Zamora J, Butler A, Whitaker H, Kote-Jarai Z, Alexandrov LB, Van Loo P, Massie CE, Dentro S, Warren AY, Verrill C, Berney DM, Dennis N, Merson S, Hawkins S, Howat W, Lu Y-J, Lambert A, Kay J, Kremeyer B, Karaszi K, Luxton H, Camacho N, Marsden L, Edwards S, Matthews L, Bo V, Leongamornlert D, McLaren S, Ng A, Yu Y, Zhang H, Dadaev T, Thomas S, Easton DF, Ahmed M, Bancroft E, Fisher C, Livni N, Nicol D, Tavaré S, Gill P, Greenman C, Khoo V, Van As N, Kumar P, Ogden C, Cahill D, Thompson A, Mayer E, Rowe E, Dudderidge T, Gnanapragasam V, Shah NC, Raine K, Jones D, Menzies A, Stebbings L, Teague J, Hazell S, Corbishley C, de Bono J, Attard G, Isaacs W, Visakorpi T, Fraser M, Boutros PC, Bristow RG, Workman P, Sander C, Hamdy FC, Futreal A, McDermott U, Al-Lazikani B, Lynch AG, Bova GS, Foster

CS, Brewer DS, Neal DE, Cooper CS, Eeles RA, Group CS, The TC. Sequencing of prostate cancers identifies new cancer genes, routes of progression and drug targets. Nature Genetics 2018;50:682-92.

9. Løvf M, Zhao S, Axcrona U, Johannessen B, Bakken AC, Carm KT, Hoff AM, Myklebost O, Meza-Zepeda LA, Lie AK, Axcrona K, Lothe RA, Skotheim RI. Multifocal Primary Prostate Cancer Exhibits High Degree of Genomic Heterogeneity. European Urology 2019;75:498-505.

10. Ewing CM, Ray AM, Lange EM, Zuhlke KA, Robbins CM, Tembe WD, Wiley KE, Isaacs SD, Johng D, Wang Y, Bizon C, Yan G, Gielzak M, Partin AW, Shanmugam V, Izatt T, Sinari S, Craig DW, Zheng SL, Walsh PC, Montie JE, Xu J, Carpten JD, Isaacs WB, Cooney KA. Germline Mutations in HOXB13 and Prostate-Cancer Risk. New England Journal of Medicine 2012;366:141-49.

11. Lange EM, Gillanders EM, Davis CC, Brown WM, Campbell JK, Jones M, Gildea D, Riedesel E, Albertus J, Freas-Lutz D, Markey C, Giri V, Dimmer JB, Montie JE, Trent JM, Cooney KA. Genome-wide scan for prostate cancer susceptibility genes using families from the University of Michigan prostate cancer genetics project finds evidence for linkage on chromosome 17 near BRCA1. The Prostate 2003;57:326-34.

12. Nicolosi P, Ledet E, Yang S, Michalski S, Freschi B, O'Leary E, Esplin ED, Nussbaum RL, Sartor O. Prevalence of Germline Variants in Prostate Cancer and Implications for Current Genetic Testing Guidelines. JAMA Oncology 2019;5:523-28.

13. Mateo J, Carreira S, Sandhu S, Miranda S, Mossop H, Perez-Lopez R, Nava Rodrigues D, Robinson D, Omlin A, Tunariu N, Boysen G, Porta N, Flohr P, Gillman A, Figueiredo I, Paulding C, Seed G, Jain S, Ralph C, Protheroe A, Hussain S, Jones R, Elliott T, McGovern U, Bianchini D, Goodall J, Zafeiriou Z, Williamson CT, Ferraldeschi R, Riisnaes R, Ebbs B, Fowler G, Roda D, Yuan W, Wu Y-M, Cao X, Brough R, Pemberton H, A'Hern R, Swain A, Kunju LP, Eeles R, Attard G, Lord CJ, Ashworth A, Rubin MA, Knudsen KE, Feng FY, Chinnaiyan AM, Hall E, de Bono JS. DNA-Repair Defects and Olaparib in Metastatic Prostate Cancer. The New England journal of medicine 2015;373:1697-708.

14. Mateo J, Cheng HH, Beltran H, Dolling D, Xu W, Pritchard CC, Mossop H, Rescigno P, Perez-Lopez R, Sailer V, Kolinsky M, Balasopoulou A, Bertan C, Nanus DM, Tagawa ST, Thorne H, Montgomery B, Carreira S, Sandhu S, Rubin MA, Nelson PS, de Bono JS. Clinical Outcome of Prostate Cancer Patients with Germline DNA Repair Mutations: Retrospective Analysis from an International Study. European Urology 2018;73:687-93.

15. Pritchard CC, Mateo J, Walsh MF, De Sarkar N, Abida W, Beltran H, Garofalo A, Gulati R, Carreira S, Eeles R, Elemento O, Rubin MA, Robinson D, Lonigro R, Hussain M, Chinnaiyan A, Vinson J, Filipenko J, Garraway L, Taplin M-E, AlDubayan S, Han GC, Beightol M, Morrissey C, Nghiem B, Cheng HH, Montgomery B, Walsh T, Casadei S, Berger M, Zhang L, Zehir A, Vijai J, Scher HI, Sawyers C, Schultz N, Kantoff PW, Solit D, Robson M, Van Allen EM, Offit K, de Bono J, Nelson PS. Inherited DNA-Repair Gene Mutations in Men with Metastatic Prostate Cancer. New England Journal of Medicine 2016;375:443-53.

16. Nombela P, Lozano R, Aytes A, Mateo J, Olmos D, Castro E. BRCA2 and Other DDR Genes in Prostate Cancer. Cancers (Basel) 2019;11:352.

17. Tomlins SA, Rhodes DR, Perner S, Dhanasekaran SM, Mehra R, Sun XW, Varambally S, Cao X, Tchinda J, Kuefer R, Lee C, Montie JE, Shah RB, Pienta KJ, Rubin MA, Chinnaiyan AM. Recurrent fusion of TMPRSS2 and ETS transcription factor genes in prostate cancer. Science 2005;310:644-8.

18. Tomlins SA, Laxman B, Dhanasekaran SM, Helgeson BE, Cao X, Morris DS, Menon A, Jing X, Cao Q, Han B, Yu J, Wang L, Montie JE, Rubin MA, Pienta KJ, Roulston D, Shah RB, Varambally S, Mehra R, Chinnaiyan AM. Distinct classes of chromosomal rearrangements create oncogenic ETS gene fusions in prostate cancer. Nature 2007;448:595-9.

19. Viswanathan SR, Ha G, Hoff AM, Wala JA, Carrot-Zhang J, Whelan CW, Haradhvala NJ, Freeman SS, Reed SC, Rhoades J, Polak P, Cipicchio M, Wankowicz SA, Wong A, Kamath T, Zhang Z, Gydush GJ, Rotem D, Love JC, Getz G, Gabriel S, Zhang CZ, Dehm SM, Nelson PS, Van Allen EM, Choudhury AD, Adalsteinsson VA, Beroukhim R, Taplin ME, Meyerson M. Structural Alterations

Driving Castration-Resistant Prostate Cancer Revealed by Linked-Read Genome Sequencing. Cell 2018;174:433-47.e19.

20. Adalsteinsson VA, Ha G, Freeman SS, Choudhury AD, Stover DG, Parsons HA, Gydush G, Reed SC, Rotem D, Rhoades J, Loginov D, Livitz D, Rosebrock D, Leshchiner I, Kim J, Stewart C, Rosenberg M, Francis JM, Zhang C-Z, Cohen O, Oh C, Ding H, Polak P, Lloyd M, Mahmud S, Helvie K, Merrill MS, Santiago RA, O'Connor EP, Jeong SH, Leeson R, Barry RM, Kramkowski JF, Zhang Z, Polacek L, Lohr JG, Schleicher M, Lipscomb E, Saltzman A, Oliver NM, Marini L, Waks AG, Harshman LC, Tolaney SM, Van Allen EM, Winer EP, Lin NU, Nakabayashi M, Taplin M-E, Johannessen CM, Garraway LA, Golub TR, Boehm JS, Wagle N, Getz G, Love JC, Meyerson M. Scalable whole-exome sequencing of cell-free DNA reveals high concordance with metastatic tumors. Nature Communications 2017;8:1324.

21. Sherry ST, Ward M, Sirotkin K. dbSNP-database for single nucleotide polymorphisms and other classes of minor genetic variation. Genome Res 1999;9:677-9.
